# Supplementary material for: Cationicity Enhancement on the Hydrophilic Face of Ctriporin Significantly Reduces Its Hemolytic Activity and Improves the Antimicrobial Activity against Antibiotic-Resistant ESKAPE Pathogens
Source: Toxins (Basel). 2024 Mar 18;16(3):156. doi: 10.3390/toxins16030156 (PMC10974533; doi:10.3390/toxins16030156)

Cationicity Enhancement on the Hydrophilic Face of Ctriporin Significantly Reduces Its Hemolytic Activity and Improves the Antimicrobial Activity Against Antibiotic-Resistant ESKAPE Pathogens

Contents of Supporting Information

Supplemental Figure S1: Correrations between net charge and hydrophobicity as well as hydrophobic moments of Ctriporin and its analogs.

Supplemental Figure S1: Correrations between net charge and hydrophobicity as well as hydrophobic moments of Ctriporin and its analogs.

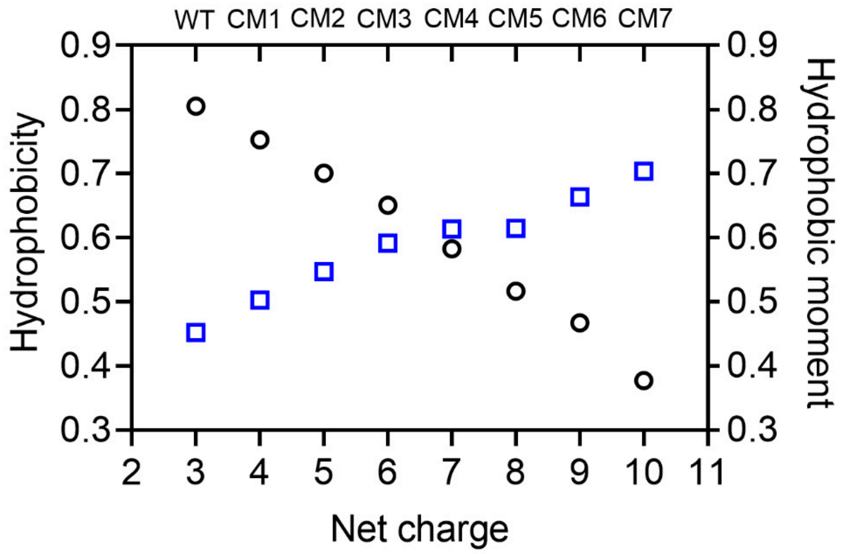

Supplement: Supplementary file 1 [file toxins-16-00156-s001.zip › toxins-2737304-supplementary.pdf]
